# Double Hydroxyl Salt as Smart Biocompatible pH-Responsive Carrier for 6-Mercaptopurine

**DOI:** 10.3390/cancers15235670

**Published:** 2023-11-30

**Authors:** Mariusz Sandomierski, Marcel Jakubowski, Maria Ratajczak, Adam Patalas, Katarzyna Gaweł-Bęben, Paulina Lechwar, Adam Voelkel

**Affiliations:** 1 Institute of Chemical Technology and Engineering, Poznan University of Technology, ul. Berdychowo 4, 60-965 Poznań, Poland; marceljakubowski1999@gmail.com (M.J.); adam.voelkel@put.poznan.pl (A.V.); 2 Institute of Building Engineering, Poznan University of Technology, ul. Piotrowo 5, 60-965 Poznań, Poland; maria.ratajczak@put.poznan.pl; 3 Institute of Mechanical Technology, Poznan University of Technology, ul. Piotrowo 3, 60-965 Poznań, Poland; 4 Department of Cosmetology, University of Information Technology and Management in Rzeszów, ul. Sucharskiego 2, 35-225 Rzeszów, Poland; kagawel@wsiz.edu.pl (K.G.-B.); plechwar@wsiz.edu.pl (P.L.)

**Keywords:** HDS, pH-responsive carrier, 6-mercaptopurine, controlled release

## Abstract

**Simple Summary:**

Drugs used to treat cancer are most often very toxic. Taking their high doses may adversely affect the patient’s health, as a result of which the therapy, instead of helping, harms. An example of such a drug is 6-mercaptopurine, described in this paper. Due to these negative properties, it is necessary to create such systems that would release the drug in a controlled manner only in the vicinity of diseased tissues. In this work, a drug carrier was prepared that releases the drug only in the acidic environment of cancer, due to which healthy tissues will not be exposed to it. Systems that release the drug only in an acidic environment have not been described before and the application potential of such a carrier is very large.

**Abstract:**

Hydroxy double salts are layered materials that are considered to be biocompatible. For this reason, research has been initiated on the possibility of their use in drug delivery. Despite their use for several types of drugs, their potential for controlled release of mercaptopurine (MERC) has not been studied. In this work, the synthesized hydroxy double salt (HDS) material was used as a carrier for this drug for the first time. The effectiveness of HDS synthesis has been proven by such techniques as X-ray diffraction (XRD), Fourier transform infrared spectroscopy (FT-IR) and scanning electron microscopy (SEM). Based on the FT-IR and energy-dispersive X-ray spectroscopy (EDS) results, the effectiveness of drug sorption was proven. The exact amount of drug retained was determined by the UV-Vis technique. The obtained results indicate that the drug is evenly distributed on the surface of the carrier, which is important during the controlled delivery of drugs. In the most important stage of the research, the effectiveness of drug release in response to changes in the pH of the environment was proven. The drug is not released into an environment that mimics healthy human tissues. It is released only after contact with the acidic environment that usually surrounds cancer cells. The low cellular toxicity of HDS and significant cytotoxic effect of HDS-MERC were confirmed by in vitro studies on MCF-7 human breast and DU145 prostate cancer cell lines and non-cancerous keratinocytes HaCaT. Interestingly, coupling with the HDS carrier increased the cytotoxic effect of MERC towards DU145 cells. Such an “intelligent” drug carrier for mercaptopurine has not been previously described in the literature. The obtained results indicate its great potential.

## 1. Introduction

Hydroxy double salts (HDSs) are layered compounds consisting of two-dimensional metal cationic nanosheets [1]. HDSs are generally stable and inert, which allows them to be used in many fields [2]. HDSs have a broad range of applications in fields such as catalysis, sensing, adsorption and ion exchange [3,4,5]. The inertness of HDSs also affects their increasing use in biomedical applications [2]. This material has been used as a drug, pesticide and bioactive ions delivery system; a material with antibacterial and antifungal properties; and as a biomolecule reservoir [2,6,7,8]. So far, these materials have been used as carriers in the delivery of drugs such as mefenamic acid, 4-biphenylacetic acid, naproxen, diclofenac, ibuprofen and valproate [2,7,8]. The use of HDS carriers has resulted in a more controlled release of drugs under certain conditions. One of the cations that can be used in the preparation of HDSs is zinc, and HDSs with this ion have been proven to be biocompatible [7].

Zinc ions are involved in many processes occurring in the human body [9]. According to the latest scientific reports, these ions have a positive effect on the osseointegration of implants [10,11]. Additionally, zinc ions play a significant role in the treatment of lymphoblastic leukemia in children. Their supplementation alleviates the effects of chemotherapy, improving the quality of life of patients [12,13]. The HDS-based drug delivery systems described so far have only been based on intercalation. Due to the strong coordination properties of zinc, an interesting direction for using these types of carriers is the formation of carrier–drug complexes, which may allow for a controlled release. One example of a drug that can be delivered in this manner is 6-mercaptopurine, which was previously delivered from a zinc zeolite carrier [14].

6-mercaptopurine (MERC) is a drug with cytotoxic, anti-inflammatory and immunosuppressive properties. The way this drug works is closely related to its dose. In high doses, it has cytotoxic and immunosuppressive effects; in small doses, it has anti-inflammatory effects [15]. Mercaptopurine is used in the treatment of cancer. It is used, for example, as an important agent in maintenance therapy in people with leukemia [16,17]. The limited use of MERC in treatment is mainly due to its poor bioavailability [18]. The low bioavailability is mainly due to the short half-life of MERC, which ranges from one to three hours. [19,20]. The disadvantages of MERC may be overcome in the future by developing appropriate drug carriers.

Various carriers for this drug have already been described in the literature. The first type of carrier described in the literature is based on the formation of disulfide bridges between the drug and the carrier [21]. Gong et al. prepared a glutathione-responsive nanoscale metal–organic framework for the intracellular delivery of MERC [22]. Talib et al. prepared MERC-coated biotinylated carbon dot nanoparticles [23]. In both works, scientists managed to prepare carriers that release MERC in response to glutathione. The release of the drug in response to glutathione allows the creation of intelligent carriers; however, if the concentration of glutathione in the cells is too low, the drug will not be released from the surface of the carrier. Other types of metal–organic frameworks have also been used for the delivery of MERC [24]. Kaur et al. used a carrier based on a zeolite imidazole skeleton, in which the drug was encapsulated inside the carrier [25]. The drug was released in response to a change in pH to acidic conditions, allowing these materials to be used to target cancer cells. Despite such promising properties, the drug was also partially released under neutral conditions, so a material that releases MERC only under acidic conditions should be applied. So far, HDSs have not yet been used for this application despite their high potential.

**Figure 1:**
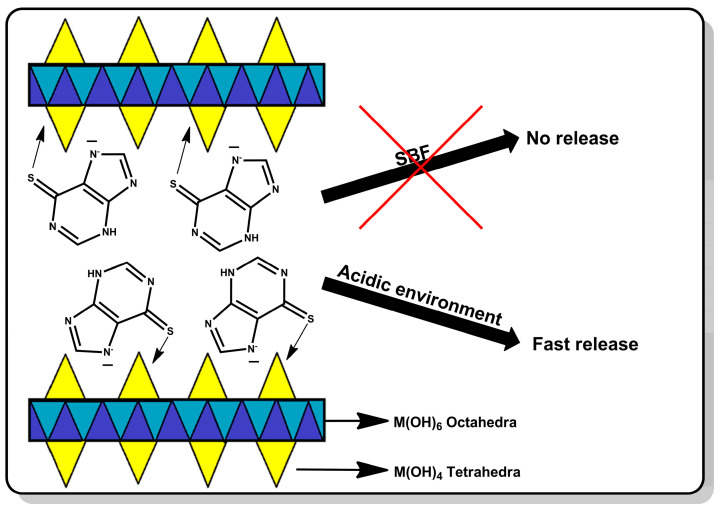
The scheme of the research presented in this work.

In this work, a smart drug carrier for mercaptopurine was prepared based on hydroxy double salts. The drug was retained on the carrier surface by the coordination interaction of mercaptopurine with the zinc ions. Due to the drug–carrier interactions, the release will be controlled, which may allow targeted delivery of the drug to cancer cells. This study aimed to prove that HDSs are capable of coordinating the drug on the surface and releasing it intelligently only under the influence of the acidic environment surrounding the tumor. The advantage of this carrier over the carriers previously described in the literature is that the drug will not be released from its surface in a neutral environment at all, which will protect healthy tissues, and the release will be rapidly induced under the influence of the reduced pH that occurs in the environment of cancer cells. The material was thoroughly characterized both before and after drug sorption. Its ability to retain the drug and release it in response to neutral and acidic environments was determined. This carrier should allow the drug to be delivered to the site of action without releasing the drug to healthy tissue. The scheme of the described studies is shown in Figure 1.

## 2. Materials and Methods

### 2.1. Materials

Magnesium chloride (Fluka, 99%) and zinc oxide (nanopowder, Sigma-Aldrich, Darmstadt, Germany) were used for the synthesis of HDS. Tris (hydroxymethyl) aminomethane (TRIS) (99.8%), sodium chloride (99%), sodium bicarbonate (99%), sodium sulfate (99%), potassium phosphate dibasic trihydrate (99%), potassium chloride (99%), glacial acetic acid (99%), sodium acetate trihydrate (99%), mercaptopurine, neutral red solution (3.3 g/L in DPBS), cell culture media and reagents were purchased from Sigma-Aldrich. Hydrochloric acid (36–38%) was purchased from Avantor Performance Chemicals (Gliwice, Poland). These reagents were used to prepare the simulated body fluid (SBF) and acetate buffer. The materials were used without further purification.

### 2.2. HDS Synthesis

During the synthesis of HDS, zinc oxide (0.5 g) and magnesium chloride (2.5 g) were used. Both reagents were placed in 5 mL of demineralized water and mixed. The mixture was stirred for 3 days at room temperature and the product was recovered by centrifugation. The white powder obtained was rinsed three times with demineralized water and then left to dry in an oven at 40 °C.

The synthesized material was named as HDS.

### 2.3. Mercaptopurine Sorption

Mercaptopurine sorption was initiated by introducing 75 mg of HDS into falcon polypropylene tubes. Each tube was filled with 45 mL of mercaptopurine solution with a concentration of 0.015 mg·ml^−^^1^ (the drug was dissolved in 0.1 M TRIS-HCl buffer at pH = 7.4). The samples were shaken for one day at room temperature; then, the samples were centrifuged. If the sample adsorbed all of the drug, it was flooded with another portion for another 24 h. The samples were flooded 6 times.

Six repetitions were performed.

The material after drug sorption was named as HDS-MERC.

### 2.4. Characterization of HDS and Modified HDS Materials

The crystallographic structure of the carrier was examined using a diffractometer D8 Advance (Bruker, Mannheim, Germany). The diffractometer was equipped with a detector (LynxEye, Bruker, Mannheim, Germany) and a Johanson monochromator. The powder was examined in the range of 2θ from 5 to 30. Samples were measured in polymethyl methacrylate cuvette. λCu Kα1 = 1.5406 Å. The technique used to characterize the carrier before and after drug sorption was Fourier transform infrared spectroscopy (FT-IR) analysis. The Vertex70 (Bruker) spectrophotometer was used during the research. The materials were tested using the ATR attachment. The drug, carrier and carrier–drug system were all characterized in the spectral range 4000–750 cm^−1^ with a resolution of 1 cm^−1^ and 48 scans. Scanning electron microscopy (SEM) images were recorded with the use of scanning electron microscope VEGA 3 (TESCAN, Brno, Czech Republic). During SEM observation, the secondary electron (SE) signals were detected. To improve the quality of performed images, all samples were coated with a thin layer (~10 nm) of gold–palladium alloy. The parameters of the measurement were as follows: magnification—20 and 50 kx, working distance—15, voltage—10 kV. SEM analysis allowed the assessment of the textural properties of the surface of the tested samples. The microscope was also equipped with an energy-dispersive X-ray spectroscopy (EDS) aperture (Bruker) which was used to determine the amount of sulfur. During the EDS test, a minimum of 10 measurements were made for each sample and the results are shown as an average value. The amount of retained and released drug was examined using UV-Vis (Shimadzu, Kyoto, Japan) spectrophotometry. During the research for this work, a UV-2600 spectrophotometer was used. Measurements of drug concentration were made in the range of 300–400 nm (λ max = 320 nm). The optical path in the cuvette was 1 cm. Calibration curves were prepared in appropriate solvents: during sorption in TRIS-HCl, during release in SBF or in acetate buffer.

### 2.5. Drug Release

The carrier (5 mg) after drug sorption was placed in 5 mL of simulated body fluid (SBF) and the released amount of drug was tested after 1 h, 8 h, 1 day and 7 days using the UV-Vis technique [14]. Due to the fact that the drug was not released, the results were not presented.

In the presented studies, a mixed environment was chosen, so at the beginning the carrier was placed in simulated body fluids (pH = 7.4) for 2 h imitating the pH of healthy tissues. After this time, the carrier was centrifuged and flooded with acetate buffer (pH = 5), which imitates the surroundings of cancer cells. After 5 min, the carrier was centrifuged and the drug concentration in the acetate buffer was assayed using the UV-Vis technique. After this step, the carrier was flooded with a new portion of acetate buffer and, after another 5 min, the concentration of the drug was tested again. These steps were repeated until all of the drug was released.

The SBF was prepared as in our previous works, and its composition is presented in Table 1 [26]. The acetate buffer was prepared according to the available literature [27].

### 2.6. In Vitro Cytotoxicity

#### 2.6.1. Cell Lines

Human breast cancer cell line MCF-7 was purchased from ATCC (LGC Standards, Łomianki, Poland); human prostate cancer cells DU145 were kindly provided by Dr Vera Knäuper, School of Dentistry, Cardiff University (Cardiff, UK); and spontaneously immortalized human keratinocytes HaCaT [28] were purchased from CLS Cell Lines Service GmbH (Eppelheim, Germany). MCF-7 and DU145 cells were maintained in Eagle’s Minimal Essential Medium (EMEM) supplemented with 10% (*v*/*v*) fetal bovine serum (FBS). HaCaT keratinocytes were cultured in Dulbecco’s Modified Eagle Medium (DMEM) containing 10% FBS. The cells were maintained at 37 °C in a humidified atmosphere with 5% CO_2_.

#### 2.6.2. Neutral Red Uptake Test

Cytotoxicity of MERC, HDS-MERC and HDS were compared in vitro using a neutral red uptake (NRU) assay as described by Repetto et al. [29]. The cells were seeded onto 96-well plates at the density of 4 × 10^3^ cells/well; grown overnight; and treated with different concentrations of MERC (10 µM, 5 µM, 2.5 µM, 1.25 µM and 0.625 µM), HDS-MERC (final concentrations of MERC in a complex: 10 µM, 5 µM, 2.5 µM, 1.25 µM and 0.625 µM) and HDS carrier (final concentrations corresponding to HDS-MERC 10 µM, 5 µM, 2.5 µM, 1.25 µM and 0.625 µM) for 72 h. Following incubation, the cells were treated with 33 µg/mL solution of neutral red dye and the morphology of the cells was documented with an inverted microscope (Nikon Eclipse, Nikon, Tokyo, Japan) connected to an Invenio II camera (DEltaPix, Smørum, Denmark). The cells were then rinsed with Dulbecco′s Phosphate Buffered Saline (DPBS) and lysed with an acidified ethanol solution containing 50% *v*/*v* ethanol and 1% *v*/*v* acetic acid. The absorbance of the neutral red released from the cells was measured at λ = 540 nm using a FilterMax F5 microplate reader (Molecular Devices, San Jose, CA, USA). The absorbance of the control cells (grown in the presence of equal volume of the solvent) was set as 100% cellular viability and used to calculate the percentage of viable cells in the other samples. Results are displayed as mean values from three experiments ± standard deviation (SD). Statistically significant differences between compared samples were calculated using one-way ANOVA and Tukey’s post hoc test by GraphPad Prism 7.0 Software.

## 3. Results

**Figure 2:**
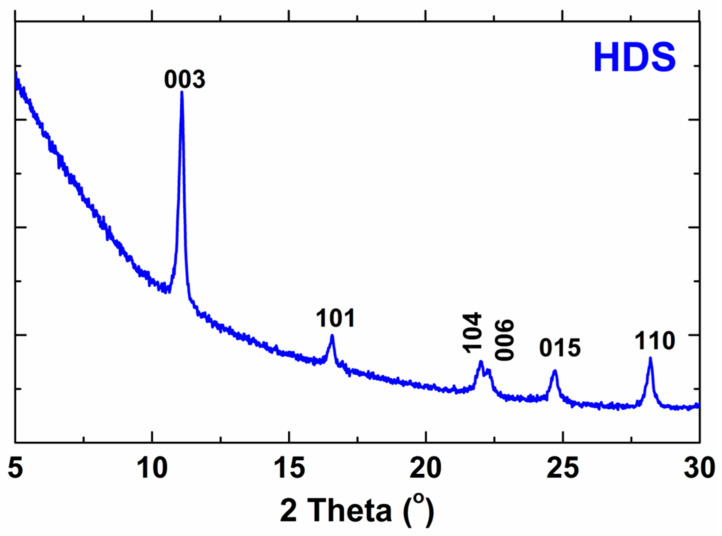
X-ray diffraction pattern of HDS.

The X-ray diffraction results, which are presented in Figure 2, confirm the successful synthesis of the biocompatible HDS. The results from the presented work were compared with the results of the reference work and summarized in Table 2 [2]. As can be seen, the results are very similar. Slight differences indicate successful HDS synthesis. The pattern of HDS indicates both strong basal reflections (consistent with the layered structure) and a series of non-basal reflections (due to the ordered arrangement of ions in the layers) [2]. The peaks presented in the XRD pattern do not occur in the structure of the substrates, which proves the reaction of the substrates.

**Figure 3:**
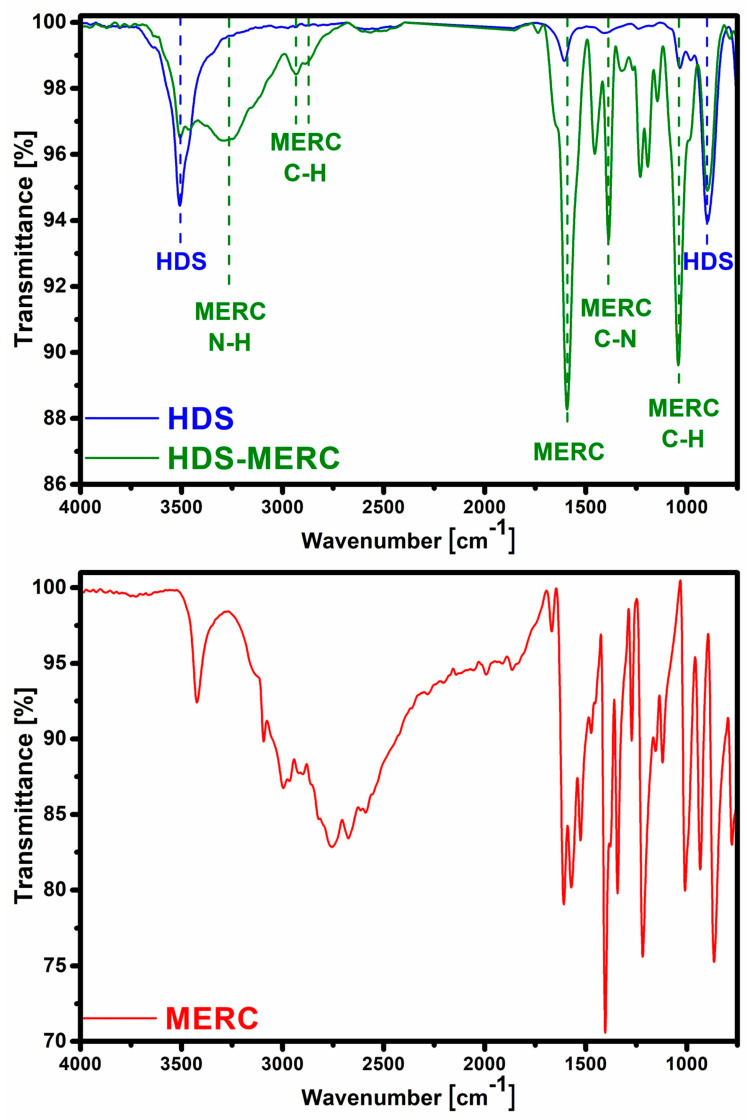
FT-IR spectra for HDS before and after sorption of mercaptopurine (**top**) and spectrum for pure drug (**bottom**).

The second technique confirming the effectiveness of HDS synthesis was FT-IR spectroscopy (Figure 3). In the HDS spectrum, there was a band at around 3510 cm^−^^1^, which can be attributed to the vibrations of the OH groups in the layers and the water molecule (both in the interlayer and adsorbed on the surface of the particles) [7,30]. The band at 1608 cm^−^^1^ indicated the presence of water molecules. The bands confirming the presence of the HDS structure were those at 1030 cm^−^^1^ and 900 cm^−^^1^ and they were assigned to the bending of M-OH groups [7].

The FT-IR technique was also used to determine the effectiveness of drug sorption. After sorption, the characteristic HDS bands at 3510 cm^−^^1^ and 900 cm^−^^1^ were still visible. This may indicate that the structure of the carrier had been preserved to some extent. After sorption, many new bands appeared, which were attributed to the presence of mercaptopurine in the material. The band at wavenumber 3265 cm^−^^1^ can be assigned to N–H stretching vibrations. Bands at 2933 cm^−^^1^ and 2878 cm^−^^1^ can be assigned to stretching vibrations of the C–H. A large band at 1590 cm^−^^1^ was characteristic of purines. The band at 1450 cm^−^^1^ can be attributed to the N-H stretching vibration [31]. The band at 1385 cm^−^^1^ was attributed to C-N bonds [32]. The band at 1038 cm^−^^1^ in the spectrum corresponded to C-H bending [31]. Another important band that appeared after the drug sorption stage was the band at the wave number of about 1270 cm^−^^1^. It belonged to the vibration of the C=S bond [33].

**Figure 4:**
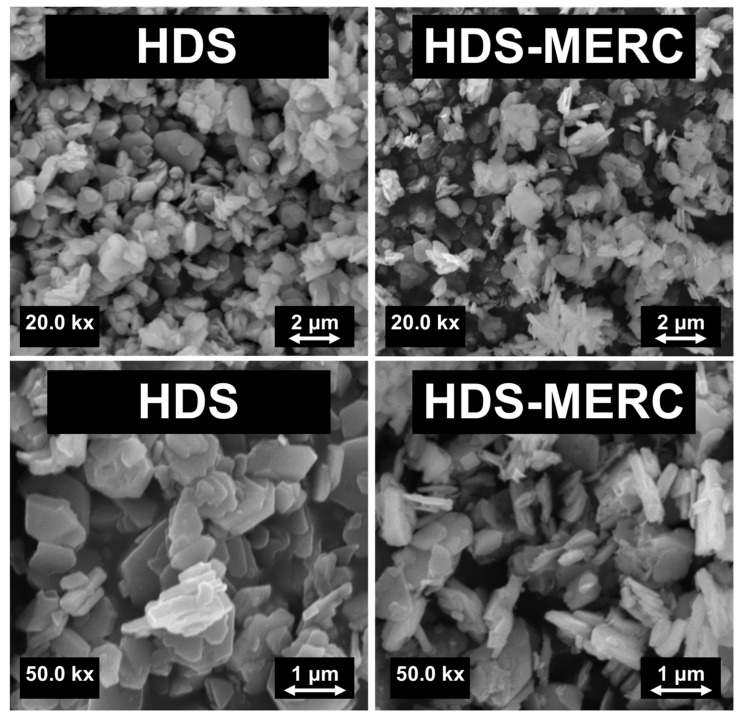
SEM images for HDS and HDS-MERC with magnifications of 20.0 kx (**top**) and 50.0 kx (**bottom**).

SEM images of the material before and after drug sorption are presented in Figure 4. These images are similar to those presented by Kaassis et al. [2]. HDS consists mainly of platelets. The morphology of the materials changes slightly after the sorption of the drug. As can be seen in Figure 4, the characteristic platelet particles that were presented for the pure HDS material were still visible but seem slightly thinner. Importantly, no additional structures were observed that would indicate drug precipitation outside the carrier surface. The material loaded with the drug also did not have such clear edges as the material without the drug. This was most likely due to a partial breakdown of the layered material. Mercaptopurine molecules get between the layers, causing their exfoliation, and thus reducing the size of the particles [34]. Unfortunately, we cannot compare these observations with other papers where HDS was used as a carrier because, to the authors’ knowledge, none of the previous papers showed SEM images after drug sorption.

**Figure 5:**
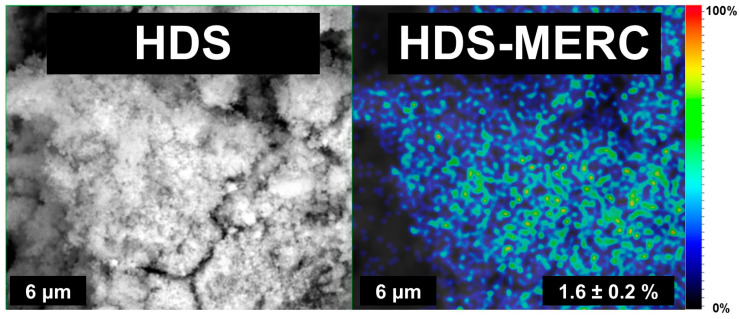
The distribution of sulfur ions, which determines the distribution of the drug in the carrier.

It is very important that, after sorption of the drug, the drug is distributed as evenly as possible over the entire surface of the carrier. Even distribution has the effect of delivering the same dose of the drug from a given amount of carrier. Uneven distribution may contribute to the release of too high doses, and thus inducing local inflammation. In this work, the distribution of the drug was investigated based on the distribution of sulfur ions. These ions were chosen because they do not occur in HDS, but occur in the structure of the drug. As can be seen in Figure 5, the ions were evenly distributed over the surface of the entire carrier. Based on this information, it can be assumed that the drug was retained only on the surface of the carrier, and not, for example, precipitated in a different form, as evidenced by the visible agglomerates of sulfur ions. The obtained EDS results indicated that the carrier contained about 1.6% sulfur, and thus about 7% mercaptopurine; however, the exact amount of drug retained was determined using the UV-Vis technique.

**Figure 6:**
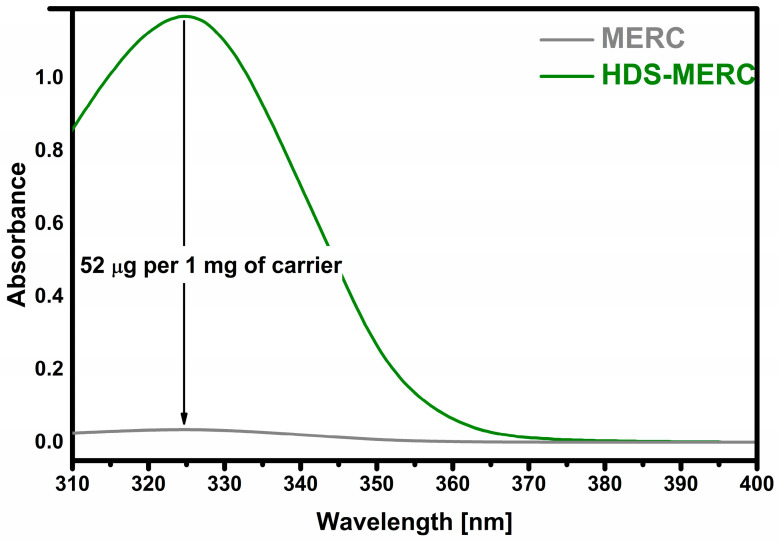
Sorption of mercaptopurine (“MERC” means starting solution).

The most important stage of the research was to determine how much of the drug could be retained and then released from potential new carriers for mercaptopurine. The sorption results are presented in Figure 6. The prepared HDS was able to retain 52 µg of drug per 1 mg of carrier which indicates a 5% loading efficiency. All drug carriers for MERC described so far have similar loading levels. A similar drug loading level was obtained for UiO-66-(SH)_2_ and amounted to 35 µg of drug per 1 mg of carrier [22]. In the case of ZIF-8 nanoparticles, the loading level was slightly higher and amounted to over 60 µg of drug per 1 mg of carrier [25]. The amount of conjugated MERC on the surface of biotinylated carbon dot nanoparticles was 5.71% [23].

**Figure 7:**
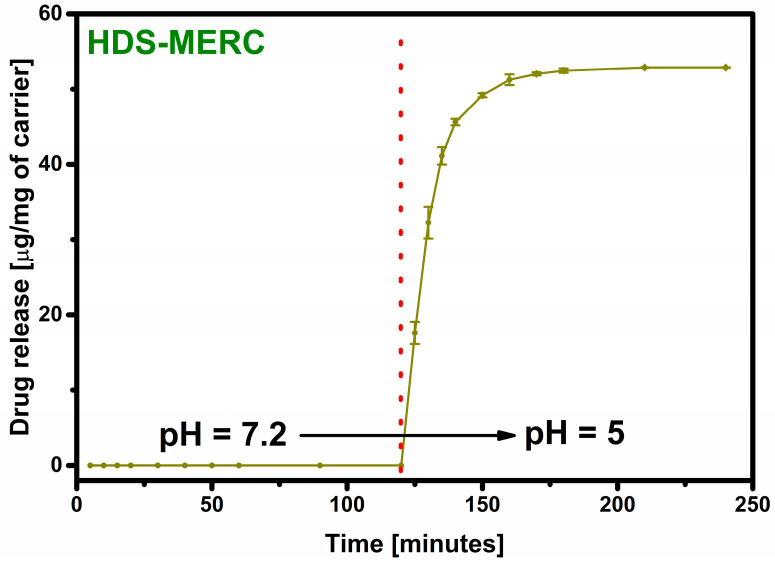
Total release of mercaptopurine from the HDS under the influence of SBF and after 120 min under the influence of acetate buffer. The red dotted line indicates the pH change.

Even when a large amount of the drug is retained in the carrier, the most important step is its release. The lack of the drug release disqualifies the material from potential use. The material prepared in this work was supposed to act as an “intelligent” drug-releasing carrier under the influence of the acidic environment that surrounds many types of cancer cells. The drug release results are shown in Figure 7. For the first two hours, the release process was carried out in a neutral pH environment imitating healthy body cells. As can be seen, under these conditions the drug was not released. The release occurred only after the pH of the environment was changed to acidic. After changing the environment, there was a large release of the drug in the first 5 min starting from the pH change (33% of the released drug). After 10 min, the amount of the drug released was over 60%, and 90% of the drug release was achieved after as short as 25 min. Such a rapid release in an acidic environment and no release in a neutral environment testifies to the very high potential of the presented material. In a way, the carrier has a protective function for the drug and does not allow it to be released anywhere other than potential cancer cells.

Comparing our material to those known by us and presented in the literature, it can be concluded that the material presented in this work has a greater application potential. A pH-responsive carrier was prepared, for example, by Mosavi et al. [24]. In those studies, it was possible to obtain a drug carrier (NMOF-5 coated with chitosan) that released higher doses under the influence of an acidic environment, but the release was longer than in the presented work. In addition, this carrier also released certain doses of the drug in a neutral pH environment, which undoubtedly shows the advantage of the material presented in this work. A similar situation was found in the work prepared by Kaur et al. [25]. The use of ZIF-8 as a carrier affected faster release in an acidic environment, but the release was not instantaneous as in this work. This material also released some doses of the drug in the SBF environment. The material presented in this paper also has an advantage over materials that are based on glutathione-induced release because its amount may often be insufficient in cancer cells to release mercaptopurine [35]. In the case of the presented material, it works only under the influence of pH, so it does not depend on the concentration of glutathione.

**Figure 8:**
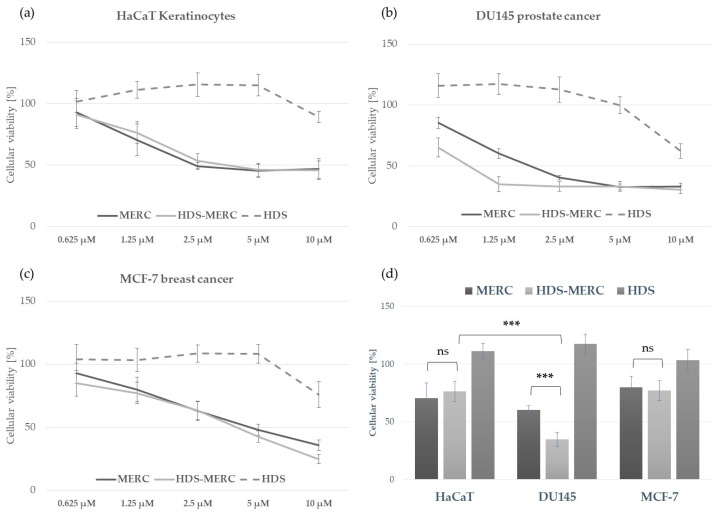
Comparison of the in vitro toxicity of MERC, HDS-MERC and HDS towards human keratinocytes HaCaT (**a**), DU145 prostate cancer cells (**b**) and MCF-7 breast cancer cells (**c**) following 72 h treatment; graphs show mean values from three independent experiments with five replicates ± SD; histogram in (**d**) presents cellular viability following 72 h treatment with 1.25 µM of MERC, HDS-MERC and HDS to emphasize enhanced cytotoxic effect of HDS-MERC for DU145 cells; *n = 3*, *** *p* < 0.001, ns—not significant.

**Figure 9:**
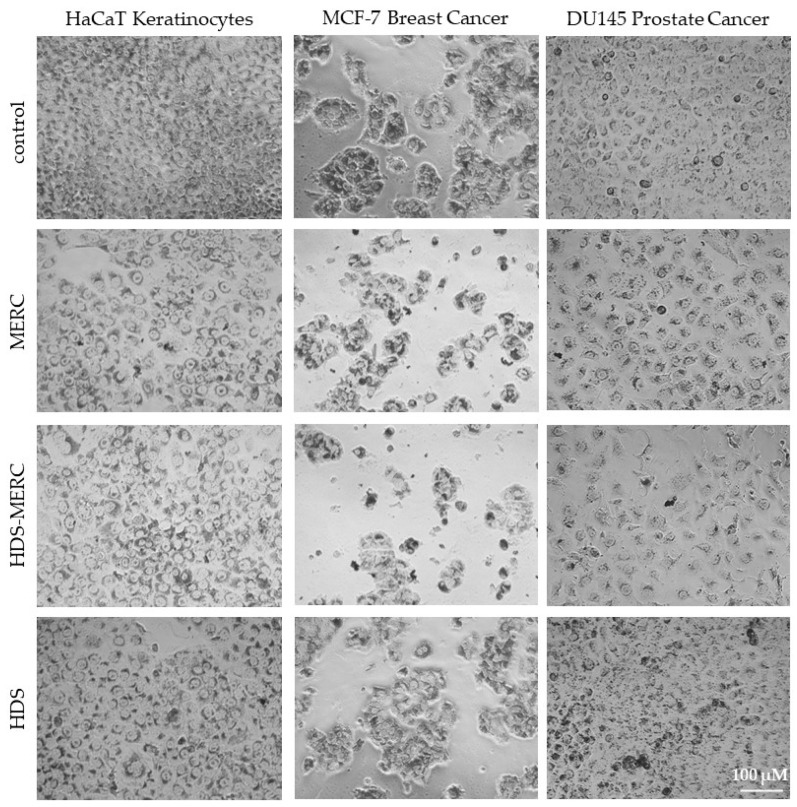
Morphology of HaCaT, MCF-7 and DU145 cells treated for 72 h with 1.25 µM MERC, HDS-MERC and HDS or appropriate solvent (control cells); neutral red staining; pictures are representative for three experiments; magnification 40×, scale bar = 100 µm.

The influence of the HDS-MERC on the viability of cancer cells was then tested in vitro and compared with the cytotoxic activities of MERC and HDS alone. For this study, we chose human breast cancer cell line MCF-7, as we have previously shown its sensitivity to MERC treatment (IC_50_ = 1.97 ± 0.51 µM) [14]; human prostate cancer cell line DU145, which is sensitive to treatment with other purine analogs [36]; and HaCaT keratinocytes as a noncancerous cellular model. As shown in Figure 8a–c, HDS was not cytotoxic for any of the tested cell lines up to the concentration of 5 µM, whereas both MERC and HDS-MERC showed significant cytotoxicity from 0.625 µM (for DU145) or 1.25 µM (for HaCaT and MCF-7). For HaCaT and MCF-7 cell lines, we did not observe significant differences in the cytotoxic effect between MERC and HDS-MERC within the tested concentration range. For DU145 cells, the cytotoxic effect of HDS-MERC at low concentrations (0.625 µM and 1.25 µM) was significantly higher in comparison with MERC. Moreover, at 1.25 µM, the percentage of viable DU145 cells was significantly lower than in the control, the noncancerous keratinocytes (34.92 ± 6.14% versus 76.40 ± 8.95%) (Figure 8d). The differences in the number of viable cells and cellular morphology between MERC and HDS-MERC treated cells were also observed microscopically (Figure 9). Cell-line-specific differences in the response to HDS-MERC and MERC treatment more likely resulted from the different metabolisms of HDS-MERC and the unique microenvironment created by various types of cancer and noncancerous cells.

## 4. Conclusions

In this work, the effectiveness of preparing the HDS material was proven. This material was used as a carrier for the anticancer drug mercaptopurine. Several techniques confirmed the effectiveness of the drug sorption. The FT-IR analysis showed that after the sorption of the drug, characteristic bands from the carrier were still visible, while SEM images showed that the carrier partially degrades. The change in the particle size of the carrier was most likely due to its exfoliation under the influence of the drug. The drug was evenly distributed on the surface of the carrier, which is important during controlled drug delivery. The prepared carrier may be loaded with the drug in a ratio of 5% to the weight of the carrier. The drug release results indicate that the carrier is an ideal candidate for pH-responsive drug release. The drug is not released at neutral pH, which is an advantage over the carriers used so far. In addition, during contact with an acidic environment (imitating tumor tissues), the drug was released almost immediately, which proves its very high potential. Very rapid release affects the delivery of a very large dose to the site affected by cancer. Materials with such a controlled release, to the authors’ knowledge, have not been described in the literature so far. HDS has not been used as a mercaptopurine carrier before, so the results obtained in this work indicate a new direction for using these prospective biocompatible materials. In vitro studies comparing the cytotoxicity of HDS-MERC with MERC confirmed that coupling with HDS might increase the toxic effect of the drug towards cancer cells, as shown for the DU145 human prostate cancer cell line. However, the enhanced cytotoxicity of HDS-MERC complexes seems to depend on the type of cells and therefore requires further investigation focusing on specific types of cancer cells and their metabolism of the HDS-coupled drug. In the next stages of research, we plan to focus on preparing a formulation that could be administered intravenously during in vivo tests.

## Figures and Tables

**Table 1:** Composition of the SBF used in this work (1000 mL of the SBF).

Order	Reagent	Amount
1	NaCl	8.035 g
2	NaHCO_3_	0.355 g
3	KCl	0.225 g
4	K_2_HPO_4_·3H_2_O	0.231 g
5	Na_2_SO_4_	0.072 g
6	TRIS	0.6112 g
7	HCl	0–5 mL

**Table 2:** Indexing of the X-ray diffraction (XRD) pattern of the obtained HDS. The results are compared with the literature.

h	k	l	2 Theta This Work	2 Theta Reference [2]	Difference
0	0	3	11.0838	11.0238	0.0600
1	0	1	16.5966	16.5760	0.0206
1	0	4	22.0110	22.0506	−0.0396
0	0	6	22.3260	22.3360	−0.0100
0	1	5	24.6689	24.6976	−0.0290
1	1	0	28.1932	28.1701	0.0231

## Data Availability

All data generated or analyzed during this study are included in this published article.
